# Nutritional Imbalances in Adult Celiac Patients Following a Gluten-Free Diet

**DOI:** 10.3390/nu13082877

**Published:** 2021-08-21

**Authors:** Aner Cardo, Itziar Churruca, Arrate Lasa, Virginia Navarro, Maialen Vázquez-Polo, Gesala Perez-Junkera, Idoia Larretxi

**Affiliations:** 1 GLUTEN3S Research Group, Department of Nutrition and Food Science, University of the Basque Country, 01006 Vitoria-Gasteiz, Spain; acardo001@ikasle.ehu.eus (A.C.); arrate.lasa@ehu.eus (A.L.); virginia.navarros@ehu.eus (V.N.); maialen.vazquez@gmail.com (M.V.-P.); gesala.p@gmail.com (G.P.-J.); idoia.larrechi@ehu.eus (I.L.); 2 Bioaraba, Nutrición y Seguridad Alimentaria, 01006 Vitoria-Gasteiz, Spain; 3 Centro Integral de Atención a Mayores San Prudencio, Ayuntamiento de Vitoria-Gasteiz, 01006 Vitoria-Gasteiz, Spain

**Keywords:** celiac disease, gluten-related disorders, gluten free diet, gluten-free products, nutritional deficiency, nutritional imbalance

## Abstract

Celiac disease (CD) is a chronic autoimmune disorder of the small intestine, whose only effective treatment is a gluten-free diet (GFD). It is characterized by the atrophy of the intestinal villi that leads to altered nutrient absorption. This study describes the nutritional imbalances which may be found in adults with CD following a GFD. During the first year of treatment, deficiencies will overcome as the intestinal mucosa recovers. Thus, biochemical data will show this progression, together with the decrease in symptoms. In contrast, in the long term, when a strict GFD is followed and mucosal recovery is achieved, analyzing nutrient intake makes more sense. Macronutrient consumption is characterized by its low complex carbohydrate and fiber intakes, and high fat (especially SFA) and sugar intakes. This profile has been related to the consumption of GFP and their nutritional composition, in addition to unbalanced dietary habits. The most notable deficiencies in micronutrients are usually those of iron, calcium and magnesium and vitamin D, E and some of group B. It is necessary to follow up patients with CD and to promote nutritional education among them, since it could help not only to achieve a gluten free but also a balanced diet.

## 1. Introduction

The incidence of celiac disease (CD) is rising all over the world. This fact is not only due to environmental factors that may decrease tolerance to gluten in diet, but also because there has been an improvement in its diagnosis [1,2]. Prevalence varies with gender, location and age and is more common in women. It has been estimated that in Western countries it is approximately 1% of the population, with a global rate of 0.7–1.4%, detected by biopsy or serologic tests [3,4]. Nevertheless, this disease is sometimes undetected, with a 1/3 to 1/5 ratio between diagnosed and undiagnosed [5]. Although the etiology of CD is not very clear, apart from genetic factors, some environmental factors can be mentioned, such as the consumption of gluten-containing cereals, infections in the first year of life or low economic status along with unsanitary environments [6].

CD is a chronic autoimmune disorder of the small intestine, which is characterized by progressive atrophy of the intestinal villi after the consumption of gluten [2,7]. The main pathological characteristics of the intestinal mucosa in celiac patients are the presence of diverse degrees of atrophy and the existence of intestinal inflammation by lymphocytes infiltrates. The degree of the damage varies between subjects, and thus patients can show a wide range of symptoms [4,7]. Although asymptomatic patients can be found, the classical form of CD presents gastrointestinal (diarrhea, steatorrhea, weight loss, etc.) and also extraintestinal symptoms like fatigue, osteopenia, iron deficiency, anemia or neurological/psychological disorders [8,9]. The immune response is triggered by dietary gluten, a protein complex present in wheat, rye and barley [4,7]. Prolamin peptides, which arise from an incomplete digestion of gluten, activate the innate and adaptive immune responses [10,11]. As a result, intestinal villi inflammation occurs and nutrients absorption is altered, leading to many deficiencies [4,7,12]. It has been seen that nutritional status of patients with CD depends on the duration of the disease without treatment, the extension and location of the lesions, and the malabsorption degree of several nutrients [13].

Nowadays, following a lifelong gluten-free diet (GFD) is the only effective treatment for this disease. Strict dietary adherence is crucial to improve duodenal mucosa and resolve symptoms [14,15]. The recovery of the intestinal mucosa takes longer in adults than in children, thus it is easier to achieve a complete recovery in children [8]. Nevertheless, even though a strict GFD is followed, the complete avoidance of gluten in the diet seems to be very difficult due to gluten cross-contamination and thus, intestinal atrophy is retained [6,8,16]. In fact, apart from the well-known wheat-based foods such as breads, pasta, pastries and other processed foods as snacks, gluten can be also found as a thickener for sauces or even as a stabilizing or flavoring additive [6]. Consequently, involuntary transgressions of gluten and consequent nutritional deficiencies among people with CD following theoretically a strict GFD is habitual [4,8].

Finally, eating habits of this collective obviously play an important role in their nutritional status. GFD should be not only gluten free but also balanced, covering all the energy and nutrition requirements. Several studies have found unbalanced profile of GFDs, characterized by low cereals, fruits and vegetables intakes and excessive of meat and derivatives [17,18,19]. Moreover, it has been reported that children and adolescents consume high amounts of specific gluten-free products (GFP). Taking into account that that these products have shown to be poorer nutritionally than their gluten-containing homologues [20,21], observed imbalances in nutrient and energy intakes could be explained, concretely increased fat consumption, which could displace fiber and complex carbohydrate intakes [17,22]. These habits can lead to many micronutrient deficiencies as well [4], less clear in adults, and thus, which need to be more deeply analyzed.

Taking all of the above into account, the need to assess the nutritional status of the celiac population is evident. Their nutritional imbalances should be detailed in depth in order to establish appropriate dietary guidelines for their correction, and so to promote their health and quality of life.

This nutritional unbalance of the GFD was also revised by Vici et al. in 2016 [17]. The present study wants to give an updated vision about this issue with results from studies published after 2015. Moreover, other important differences between these two reviews need to be pointed out. First, whereas Vici et al. described the nutritional deficiencies among celiac children on a GFD, the present review studies in children (as well as those in the general population) have been used for comparison of results obtained in celiac adults. Secondly, the present study shows results related to the dietary adherence of people with celiac disease. Finally, the present review provides the most important dietary guidelines to achieve the nutritional balance with a GFD, so that it could represent a useful tool of nutritional education for dietitians and nutritionists working in the field of celiac disease.

The aim of this review is to describe the nutritional imbalances, deficiencies and excesses, of celiac adults who follow a GFD, at the beginning of treatment and once established, in the long term, and to compare variations of these imbalances between men and women. The dietary profile between celiac patients and the general population is also compared in order to underline the added difficulty of eliminating gluten from the diet and its impact on the observed deficiencies. Taking all into account, some dietary recommendations are suggested.

## 2. Materials and Methods

PUBMED database was searched for articles published since 2000, and using different combinations of the following terms: celiac disease and gluten-free diet and dietary deficiencies, nutritional deficiencies, nutrient intake, micronutrient, macronutrient, vitamin, mineral, or fiber intake.

Inclusion criteria were as follows: observational studies, case-control studies, cohort studies and systematic reviews were included. Only studies with participants following a GFD and with nutritional assessment in terms of macro- and/or micronutrients, fiber, and/or biochemical data from participants were included. Papers with information on at least one nutrient were included. The selection process of articles regarding the nutritional deficiencies of adult celiac people following a GFD is shown in Supplementary Figure S1.

Nevertheless, along the text some other important studies have been mentioned, in order to clarify, explain or justify some of the observations extracted from selected articles, based on authors’ experience (articles concerning deficiencies but in celiac children, articles related to the nutritional composition of GFP, to the dietary adherence, etc.).

## 3. GFD in CD Treatment

### 3.1. Newly Diagnosed Patients: Recovery of Previous Nutritional Deficiencies

As previously mentioned, it is well known that untreated celiac patients, as well as those newly diagnosed, present many serious nutritional deficiencies. GFD introduction ameliorates these shortages that are related to the progressive recovery of the intestinal mucosa, which, in turn, depends on the person, the duration of the disease untreated, the severity of mucosal lesions, etc. [23,24,25]. Table 1 presents data from the selected studies on the nutritional status of people with celiac disease.

Once GFD is established, nutritional deficiencies are not reversed quickly, they improve gradually and sometimes do not even normalize. Regarding iron deficiency, Annibale et al. [24] observed that during the first year of GFD, anemia improved notably in most cases (but not in all) due to the recovery of the intestinal mucosa. Recovery in women appeared to be slower than in men because of menstrual blood loss [26]. Likewise, other authors observed impairment of several nutritional indices, like hemoglobin and folate, after one year of GFD [27]. Thus, in newly diagnosed cases, apart from assessing GFD, it might be interesting to treat deficiencies with supplements, such as iron, especially in women. Afterwards, when normal values are reached and mucosal recovery is supposed to be achieved, a GFD on its own might be enough.

Similar results are found when analyzing vitamin D deficiency and bone structure parameters. Zanchetta et al. [28] and Sategna-Guidetti et al. [27] described that bone microarchitectural parameters and vitamin D levels improved in celiac adults in their first year on a GFD. Nevertheless, these parameters were still low, and also lower than in healthy controls, even after taking supplements. In fact, vitamin D levels did not reach 30 ng/mL, and it is important to note that PTH activity is decreased and not stabilized until this values of vitamin D are achieved [29]. These data agree with those presented in a study from Stenson et al. [30], where CD prevalence was much higher in people with osteoporosis and showing low vitamin D levels. Thus, to face vitamin D deficiencies in celiac patients during the first year on GDF it would be interesting to supplement vitamin D and recommend suitable sun exposure [31].

As mentioned, the recovery of nutritional deficiencies is related to the amelioration of the intestinal mucosa due to the removal of gluten from the diet. However, not all patients show mucosal recovery after one year of GFD [27]. Lanzini et al. (2009) suggested that a complete normalization of duodenal lesions is exceptionally rare in adult celiac patients [32]. Similarly, Tursi et al. (2006) observed that older adult patients (>30 years) show incomplete endoscopic and histological recovery even 24 months after starting a GFD [33]. Nevertheless, it has been proposed that a correct adherence to the GFD leads to an improvement of the intestinal mucosa and symptoms reduction in about 6–12 months [23,24]. These discrepancies could be due to differences in compliance with the GFD. On the one hand, some patients admit to take gluten containing foods from time to time [32]. On the other hand, although it is common to find celiac patients who claim to follow a completely strict GFD, the lack of mucosa recovery could indicate that they may involuntarily consume gluten. For this reason, analyzing dietary adherence together with the dietary habits of this collective is crucial.

### 3.2. Adherence to the GFD

It has been estimated that gluten transgression in celiac population is very frequent, between 36 and 55% [34]. It is noteworthy that Kurppa et al. claim that adherence to the diet can be achieved; in Finland they obtained 88–90% adherence values in adults due to the high prevalence and good knowledge of CD in the country [35]. Even so, some authors suggest that GFD adherence observed in studies may not be representative of total celiac population, since participants in clinical trials are probably more aware of following a correct GFD, and also because those that take part in studies are very strict [36].

Many factors have been associated with lower diet adherence or occasional gluten intakes, such as: young age at diagnosis, adolescence, local food culture, lower socioeconomic status, travelling and eating in restaurants, the absence of symptoms at present (asymptomatic patients may have higher occasional gluten consumption [6,35]) and low degree of knowledge or motivation of the patient [6].

Another factor that must be taken into account in celiac patients is the quality of life, since it has been seen that they tend to have a lower perception. After diagnosis, this usually improves with GFD treatment because, symptoms are reduced [37,38]. Even so, after about one year of treatment life quality remains lower, which can be explained by the restrictions and limitations of following this diet [6]. In addition, there are some patients who find so difficult to follow this type of restrictive diet that they tend to seek alternative therapies [39]. Serial endoscopies with collection of duodenal biopsies monitor the effectiveness of the GFD. To assess the compliance with the GFD several procedures are employed, alone or in combination, such as periodic visits to nutritionist, clinical follow up, the use of structured questionnaires, serological controls of specific antibodies and the determination of gluten peptides derived from gluten in feces and/or urine [40]. Appropriate detection of dietary transgression could help to predict indirectly that sufficient recovery of the intestinal mucosa is not achieved. Nevertheless, it must be pointed out that the intestinal mucosa structure recovery neither ensures a normal intestinal function at molecular level nor the expression of some genes necessary for the absorption of some micronutrients [41,42].

### 3.3. Nutritional Composition of the GFD

Studies on nutritional deficiencies in the first year of GFD show some possible deficiencies based on biochemical data, but only a few nutrients are described. Once the mucosa is recovered, it is assumed that a strict diet without gluten has been followed in a long term and thus, measuring nutrient intake in celiac people to assess their nutritional status, with supposedly no absorption problems, makes sense [43].

#### 3.3.1. Macronutrient Intake

Macronutrient intake and distribution in GFD is presented in Table 2. Various imbalanced patterns are repeated across different studies, which have been carried out in people from several countries who followed GFD in different durations.

(a) Fats

All studies agree that fat intake of celiac adults is unbalanced. Some of them only show high fat intakes [19,25,47] but others also observe a high consumption of saturated fatty acids (SFA) or an excessive intake of cholesterol [17,18,36,48]. These results do not differ from those obtained in children, who present high fat intakes, with increased SFA/polyunsaturated (PUFA) ratio [4].

This could be due to the low intake of plant-based foods and high consumption of processed GFP [19,44]. When people with celiac disease follow a GFD, it is common to consume GFP, and these tend to be generally higher in total and SFA than their gluten containing analogues [4,20,21]. Additionally, Wild et al. compiled some records of GFD and described that 47% of the energy intake came from processed products. Therefore, correct classification of GFP is needed, so that celiac patients could be more informed and choose these products appropriately [44].

Unbalanced diets rich in SFA lead to health problems such as increased risk of cardiovascular diseases (CVD), or insulin resistance (IR) in all individuals, general and celiac population [49,50,51]. In fact, it has been observed that people with CD show higher risk of death from CVD [52]. In addition, the consumption of processed products has been related to higher mortality [4,53]. Another common problem is the chronic low-grade inflammation which has also been related to this context of an unbalanced diet with the aforementioned characteristics [54].

(b) Carbohydrates

When people with CD start on a GFD they have to stop consuming gluten-containing cereal based foods, which are the most commonly consumed cereals. Moreover, cereals are the basis of a balanced diet, and thus, without some suitable guidelines diet can result imbalanced [23,55].

As it can be observed in Table 2, several studies have shown that patients who follow a GFD present low carbohydrate intakes [18,19,25,45,47,48]. This may be due to their fear about consuming gluten that makes them reject cereals. To be more precise, celiac patients do not consume enough complex carbohydrates. Thus, Wild et al. [44] observed a low complex carbohydrate intake; however, total carbohydrate intake seemed to be enough due to the high consumption of simple sugars and processed food. This is consistent with the fact that GFPs tend to have a higher glycemic index than their gluten containing counterparts [50,56]. Thus, it can be assumed that the imbalances observed are not only due to the low or non-existent consumption of gluten-containing cereals, but also to the high intake of processed GFP, and the low consumption of vegetables and legumes [44].

When comparing these data to that of children, similarities can also be observed. Children present a low consumption of foods rich in complex carbohydrates, although the intake of simple carbohydrates is higher due to GFP [4].

This dietary profile with high intakes of simple sugars and high-glycemic index products could also be harmful; they could cause the aforementioned IR, which is associated with the more frequently observed hyperinsulinemia, lower glucose tolerance and increased risk of diabetes [51,57].

(c) Fiber

Foods that are high in carbohydrates are usually also rich in fiber [52], but are unusual in the diets of celiac patients [17,19,44]. Most studies evaluating nutrient intake in GFD show low fiber intake, which is in perfect agreement with the low CHO and high fat intakes mentioned above [18,19,36,44,45,46,47,48]. Regarding the intake of fiber in children, no differences are found either. This low fiber intake also seems to be explained by the low consumption of fiber-rich plant foods and whole-grains [55], and by the high consumption of refined processed foods [4].

Low fiber intake has been linked to a higher prevalence of constipation and increased risk of diverticulitis. Moreover, it has also been related to an increased risk of gastrointestinal symptoms commonly present in CD and even in treated celiac patients [58]. Thus, even though a direct relation between GFD and constipation or diverticulitis has not been observed, it could be thought that higher fiber intakes among these patients could help to improve the inflammation that is noticeable in the disease [59,60], and reduce symptoms such as abdominal pain [61].

(d) Proteins

Some studies, like those of Martin et al. [45], González et al. [18] and Ballestero-Fernández et al. [48], claim that the protein intake is higher than recommended in celiac patients who follow a GFD, probably due to excessive meat intake. However, other studies do not highlight the same results and neither do the ones obtained in celiac children who follow a GFD. In fact, some studies show contrary data, higher protein consumption among non-celiac children [4,12,62].

#### 3.3.2. Micronutrient Intake

As for macronutrients, the studies listed in Table 2 show that the intake of vitamins and minerals is also impaired. On the one hand, it must be mentioned that most studies do not measure the intake of all micronutrients, so precise and exact conclusion cannot be done [36,43,46]. On the other hand, it is important to take into account that depending on the reference intakes of each country controversial results can be found when defining the deficiencies of each nutrient.

(a) Vitamins

When analyzing vitamin intakes, there are several studies that show deficiencies for the same vitamins, such as that of vitamin D and vitamin E [18,19,25,44,48] followed by low intakes of B group vitamins like folate (B9), thiamine (B1), riboflavin (B2), and pyridoxine (B6) [17,18,19,25,43,44,45,55].

Vitamin D deficiency may be of special importance, since a higher prevalence of osteoporosis has been seen in people with CD, and this vitamin is considered of vital importance in bone metabolism [30,31,63]. In addition, as above mentioned, its supplementation has been recommended during the first year of GFD in order to recover the nutritional deficiencies due to low absorption caused by the pathology.

Regarding B group vitamins, the observed deficiencies agree with the biochemical data presented by Hallert et al. who observed low B12 and low folate biochemical levels and declared that homocysteine (tHcy) levels were raised in these patients, higher than in the general population, even when following GFD for a long time [43,64]. It is well known that high tHcy levels are linked to increased risk of CVD [43,65], and, as mentioned before, CD has higher prevalence of this disease [51]. To face this problem, it is important to highlight that the deficiencies mentioned have been associated to a low intake and not to the intestinal malabsorption [43]. Moreover, it has been observed that high tHcy levels can also be normalized thanks to a supplementation of B group vitamins. Thus, dietary treatment and appropriate follow-up are especially important [18,36,44] to mind these aspects too. Di Nardo et al. attached more importance to folate intake, so they recommend consuming pseudo-cereals which are richer in this vitamin, such as quinoa and amaranth [4], apart from its typical consumption through vegetables and pulses.

In addition, low B group vitamin levels have been reported to be associated with a worse sense of quality of life [43], and their supplementation with better general well-being [64].

Deficiencies in B vitamins and vitamin D seem to be common in children with CD too but that of vitamin E is not mentioned [4,43].

(b) Minerals

In general, the most common mineral deficiencies described in the literature are those of iron, calcium and magnesium [17,18,19,25,36,44,45,46,47,48]. Deficiencies in iodine, potassium and zinc can also be found [18,19,25,44,45]. Finally, there are some studies which present low intakes of selenium, sodium and manganese [19,25,44].

Some of them can be normalized with a suitable GFD-based treatment, such as in the case of zinc deficiency. Nevertheless, GFD may not be enough to overcome other ones, such as magnesium deficiency, due to the fact that cereal-based GFPs have lower mineral content than their gluten-containing analogues [66]. Special attention should be paid to iron deficiency. This is a major problem in non-treated active celiac disease and in patients with incompletely regenerated mucosa who have difficulties in achieving normal iron values [67]. Hallert et al. [43] did not see serum iron deficiencies after 10 years of GFD; since ferritin levels were within normal, but female celiac patients showed lower ferritin levels than male patients [44,46,48]. In addition, iron deficiency has been declared as a common complication of CD, so these low levels should be considered alarming, especially in women [45]. Moreover, it can worsen because of the observed low consumption of legumes and cereals [18,19].

In relation to calcium, controversial results can be found depending on the reference intakes of different countries. Hopman et al. observed a low intake of calcium according to the American recommendations (ARDA) and to Moreiras et al. [55] but adequate results with regard to Dutch recommendations [36]. On the one hand, Thompson et al. reported that 19% of their patients were lactose intolerant and 34% had been intolerant previously. So low calcium intake could also be related to low dairy intake of these patients, or even to the fear of feeling bad or causing harm [46]. Furthermore, although calcium intakes are similar to the general population, it must be taken into account that in recently diagnosed celiac patients too little lactase is produced due to the damaged mucosa and thus they develop a secondary lactose intolerance. Even though this alteration improves with the regeneration of the mucosa, calcium intake together with appropriate levels of vitamin D may benefit newly diagnosed patients, [27,28,45]. Therefore, when meeting patients with lactose intolerance, which seems to be frequent in CD, calcium supplementation could be considered, since dairy consumption will be greatly reduced or avoided [45].

Regarding mineral deficiencies in children, the most described are those of iron, calcium, magnesium and zinc. Even so, and unlike in adults, deficiencies of potassium, iodine, selenium or manganese are not mentioned [4].

Table 3 provides a summary of nutritional deficiencies in adults and children with celiac disease.

It must be pointed out that, according to Larretxi et al., it seems that the influence of GFP on micronutrient deficiencies is limited, since GFPs and their gluten analogues do not contain large amounts of typically lacking micronutrients. Therefore, these deficiencies could be more related to an unhealthy lifestyle: low vegetables, fruits, cereals and nuts intake followed by high meat consumption [67]. Thus, recommendations to correct these mistakes and to promote healthy GFD should be given to amend those habits.

Nevertheless, it is noteworthy that wheat flour and its derivatives are usually fortified with some micronutrients, such as iron or folic acid, but no other alternative flours, like those used in GFPs [4,22,68,69]. Thus, taking into account observed nutritional deficiencies, the fortification of GFPs could be a matter of interest.

Finally, taking into account that some patients may continue having a suboptimal intestinal absorption throughout their lives, the recommended intakes for the general population may not be valid for all celiac patients, so that it might be more appropriate to establish reference intakes for the celiac population that follows a GFD. Even so, it would be necessary to evaluate individually these patients through biochemical analyses and combine it with the dietary record, in order to give more personalized and effective treatment recommendations [32,45].

#### 3.3.3. Differences between Men and Women

In general terms, unbalanced macronutrient intake and distribution of women and men are quite similar [18,19,44]. Nevertheless, although both genders present a low fiber intake [45], it has been observed that there are fewer women who reach a suitable consumption [18,19,46].

Regarding micronutrient deficiencies, differences among genders have been described. Among vitamins, intake does not vary between genders [18]. Nevertheless, some specific differences can be mentioned, although these are not always observed. Jamieson et al. registered low intake of vitamin C in men, but not in women [47]. Moreover, sometimes contrary results are appreciated, as in the case of B vitamins. One study detected that folate deficiencies were somewhat higher in women [44], while another described more serum folate and pyridoxal 5′-phosphate deficiencies in men [43]. Similarly, Hallert et al. described higher tHcy levels in males than in females, despite being high in both genders. These results have been related to higher vitamin B deficiencies and agree with the fact that celiac men tend to consume 50% less folate-rich foods than celiac women.

In terms of minerals, while women have a better fulfillment of dietary magnesium requirements, men have a better adherence to iron, iodine, potassium and calcium intakes [18,19,43,44,46]. Nevertheless, there are studies that do not find significant differences between men and women either for these or for other minerals that usually present differences, such as magnesium or calcium [45]. Finally, controversial results can also be found, such as for the intake of selenium, which have been shown to be both higher [19] and lower in men [44] than in women in various studies. Table 4 summarizes aforementioned differences between men and women.

Lower intake of micronutrients among women could be because they normally tend to consume lower amounts of food and total energy. Even so, it is important to note that most of the patients participating in the studies are usually female [25,36,44,45,46,48] as can be seen in the studies included in Table 2. Thus, it can be suggested that the general summary of the GFD present in this review is more representative of the female gender. Therefore, more studies with a greater number of male patients are needed, in order to properly compare the diet quality among genders.

### 3.4. Comparison with the Healthy General Population

It is of great interest to compare the GFD model with the diet of the general population in order to be able to determine whether the imbalances in the GFD are due exclusively to the difficulty of eliminating gluten from the diet, or if unhealthy habits also influence.

Concerning energy intake, some authors claim that celiac patients tend to have lower energy intake [19,45], while others describe that is higher because of a higher consumption of processed products [44]. Nevertheless, in general terms, differences in energy intake are not usually observed between celiac patients and the healthy general population [18,36].

#### 3.4.1. Macronutrient Comparison

Following on with macronutrient consumption, it has been observed that celiac people who follow a GFD tend to consume too much fat [18,45], but this imbalance is similar in the general population [18,44,48]. Nevertheless, literature comparing these intakes between celiac people and the general population shows that lipid consumption can differ between both groups. Some authors have described that general population presents a higher fat intake than that on a GFD [19]. By contrast, other show a higher fat intake in celiac patients than in the general population [45] and, finally, Hopman et al. found no differences in terms of the total amount of fat, but higher SFA in people on a GFD [36]. These differences could be explained in terms of the magnitude of the consumption of specific GFP products. Taking into account that the composition of these products is higher in fat (used for substituting gluten), it increases fat consumption [4,20,45].

Although different studies have indicated the low carbohydrate intake in the GFD of celiac [18,19,25,45,48] no differences are found with the intakes of the general population [18,19,48]. However, Martin et al. report a slightly lower complex carbohydrate intake in celiac patients than in healthy adults [45]. Also higher intake of simple sugars can be found in celiac patients who follow a GFD [44].

Similarly, previously mentioned low fiber intake is also observed in both groups. Some registered values indicate a slightly higher fiber intake in the general population, although it was still low [18,19,44].

Finally, although a higher protein intake has been reported in the celiac population [18,45], no usual differences with the general population have been seen [18,19,48], which means that both diets are hyperproteic.

#### 3.4.2. Micronutrient Comparison

Regarding the differences in micronutrient intakes, similar deficiencies are usually found in controls subjects [18,19,36,44,48]. Even so, differences have been recorded in some cases. Lower intakes of vitamin E, niacin, iron and magnesium in celiac patients than in the general population have been described, but higher ones of riboflavin, B6, zinc and potassium [18,19]. Controversial results can be found with regard to selenium, folate and B group vitamin intake [19,44,45], observing both higher and lower intakes depending on the study. There are not enough studies that compare micronutrient intake between celiac and general population, thus it is difficult to establish conclusions. Nevertheless, although in some cases higher intakes of these vitamins have been seen in celiac patients, most studies agree that the intakes are usually lower in the patients who follow a GFD [43,44,45,64].

This is consistent with the lower blood levels of B vitamins and higher tHcy levels that have been found in celiac people, compared to healthy subjects. Moreover, this can be explained, at least in part, by the fact that gluten-free cereal products contain lower amounts of folate [43], as previously mentioned, so that folate intake through bread is higher in the general population than in celiac.

Despite the mentioned results, the problem to highlight is that there is a nutritional deficit of micronutrients in both, celiac and general population. Moreover, some authors claim that celiac do not seem to be at an increased risk of some deficiencies, such as that of B group vitamins, comparing with general population, since no significant differences have been observed in the percentage of participants not reaching reference values [45].

Considering all the above, and according to González et al., the poor macronutrient distribution and the micronutrient deficiencies observed in the GFD could be related, more than to the consequence of eliminating gluten from the diet, to geographical dietary habits: low fruit, vegetables and cereal consumption and high intake of meat. In fact, the same unbalanced dietary patterns are observed both in celiac and in the general population from the same countries [18,19,55]. Thus, all individuals in the population need dietary advice, not only the persons with celiac disease. It will be more appropriate to give general recommendations to promote the intake of food rich in micronutrients to improve these deficiencies in both, celiac and general population [43]. And in the case of celiac people food that naturally do not contain gluten should be consumed.

Even so, it would be interesting to analyze more biochemical data to compare them with the dietary intakes and thus, to identifying direct interactions between low nutrient intakes and health. It is also important because although micronutrient intakes are enough, the intestinal mucosa is not always recovered, as mentioned above, which could lead to low micronutrient serum levels [45]. Moreover, participants in studies may change their eating habits during the food intake recording period. Therefore, dietary data from those trials may not show the reality, and serum values could help to get closer to the true data [43,46]. It must be also pointed out that most of the studies obtain control intake from national dietary surveys, and it would be desirable to have better age- and gender-matched non-celiac adult controls.

In addition, those studies that measure micronutrient intake of general population show different results, and thus the comparison between them and those of the celiac population can vary. For example, in a study made by Wild et al. celiac females had a higher intake of magnesium and calcium than healthy women, or by contrast lower intakes of magnesium, iron, zinc, manganese, selenium and folate, depending on the control data selected as a reference [44].

Table 5 summarizes the differences between coeliac patients and the general healthy population found in the present review.

### 3.5. Dietary Guidelines for a Balanced Diet

Professional nutritional counselling by nutrition adviser is highly desirable from the beginning of the gluten free therapy. It is necessary to follow up patients with CD and to promote their knowledge in relation to the pathology and GFD, especially if symptoms or deficiencies regarding micronutrients persist, since it could help to improve adherence to GFD, and therefore to achieve a gluten free and balanced diet. This practice would lead to a correct recovery of the intestinal mucosa and to a healthy nutritional status. In fact, an adequate and varied GFD, enriched in fruits, vegetables, and fibers, does not necessarily lead to malnutrition. A recent study by Gładyś et al. (2021) evaluated the impact of standard dietary education in adult celiac patients on a GFD, by measuring the nutritional composition of the diet before and after one year of the education. Results showed that although adherence to the diet was improved, nutritional profile of the GFD did not, revealing the necessity to increase the role of dietitians in the treatment of CD [70].

- The most remarkable guideline would be to improve the diet by promoting a greater consumption of plant-based foods, such as fruits, vegetables, legumes, nuts and naturally gluten-free whole grain cereals and pseudocereals followed by reducing GFP consumption.

Regarding macronutrients, these will help to reduce the intake of poor-quality fats and simple sugars, and at the same time help to increase the consumption of complex carbohydrates, fiber, vitamins and minerals [18,19,55].

The consumption of pseudo-cereals, such as quinoa and amaranth, is also an excellent option, since they are considered good sources of some micronutrients such as folate, riboflavin, vitamin C and vitamin E, and they are also not as expensive as GFP [4,55].

- Although we can consider that protein intakes are sufficient, we will have to make sure they understand the importance of protein sources by promoting the intake of high-quality protein rich foods, which will be also related to the intake of high-quality fats, and thus a better dietary lipid profile [55].
- Naturally gluten-free foods rich in micronutrients are proposed before recommending fortified foods or supplements [55]. However, it could be interesting to combine the two options, with the aim of achieving a faster recovery from some vitamin and mineral deficiencies, thus, once suitable levels have been recovered, it could be enough to follow an appropriate GFD.
- Another possible improvement to consider is the one mentioned by González et al. who claim a fortification of the GFP, knowing in advance which are the nutrients that are most needed in the GFD [18]. This, could help to improve micronutrient deficiencies, but on the other hand, the macronutrient content should be corrected (reducing fats for example) and thus total balance could be achieved. In fact, it is noteworthy that the food industry is making great progress in developing healthier GFPs, which is a great challenge that has a direct impact on the health of the patients.
- The deficiencies in iron, calcium and vitamin D are noteworthy, in relation to their involvement in pathologies such as anemia or osteoporosis, which are more prevalent among the celiac population. Thus, it is recommended to take care of their intake through their food sources, such as legumes, cereals and dairy products [55].
- Similarly, to overcome deficiencies observed in B group vitamins, involved, at least in part, in the higher prevalence of CVD in CD, dietary treatment is especially important. Folate is usually given more importance; its consumption should be promoted through vegetables, pulses and pseudocereals. Moreover, this micronutrient is one of those proposed for food fortification and liable to be obtained through supplementation in high risk cases [18,36,44].
- It is common to find nutrient deficiencies in GFD, but this does not mean that it has to be normalized. Thus, Bascuñan et al. claim that although adherence to GFD may seem enough, patients with celiac disease should be continuously supervised to prevent some usual deficiencies, and to ensure that they continue having sufficient adherence to the GFD [25].

Considering what has been described so far, the proposed dietary recommendations for a balanced gluten-free diet and their result are given in Table 6.

## 4. Conclusions

GFD should guarantee the absence of gluten to progressively recover intestinal mucosa at the beginning of the treatment and consequently the nutritional deficiencies caused by the pathology. In the long term, assuming that a strict diet is being followed and mucosa is recovered, the dietary balance should be the goal to achieve, which is possible through a correct and varied GFD. However, GFD is usually unbalanced for macro and micronutrients, in both, celiac women and men. This is mainly due to unhealthy dietary habits, commonly found as well in the general population, but also to the difficulty of eliminating gluten from the diet, that leads to a low cereal intake of cereals and high consumption of processed GFPs.

Taking everything into account, it is vital to carry out a continuous and personalized follow-up of celiac patients from the moment of diagnosis. For this supervision, the presence of a nutritionist is essential, so that these patients could obtain proper nutritional education and thus strictly adhere to the GFD, which will be the key for the long-term balanced diet.

## Figures and Tables

**Table 1:** Nutritional status of people with celiac disease following the first year GFD: biochemical data and anthropometric parameters.

Author	Sample Size (n) GFD Duration	Type of Study	Country	Biochemical Data	Anthropometric Parameters
Zanchetta et al. (2017) [28]	−n = 26 (women) −1 year GFD	Observational, longitudinal cohort study	Argentina	Low vitamin D Normal Ca, Hb, PTH	Low bone microarchitectural parameters
Annibale et al. (2001) [24]	−n = 20 −GFD: 6, 12 and 24 months		Italy	Low Fe, low Ferr, low Hb Anaemia Normal Glu, TG, proteins, Alb	
Sategna-Guidetti et al. (2000) [27]	−n = 86 −1 year GFD		Italy	Low vitamin D Normal Ca, P, Alb, PA, Hb, Fe, Ferr, Trans, Fol	BMI = 20.85 kg/m^2^

GFD: gluten-free diet. Ca: calcium. Hb: hemoglobin. PTH: parathyroid hormone. Fe: iron, Ferr: ferritin. Glu: glucose. TG: triglycerides P: phosphate. Alb: albumin. PA: pre-albumin. Trans: transferrin. Fol: folic acid. BMI: body mass index.

**Table 2:** Dietary profile of people with celiac disease following a GFD.

Author	Sample GFD Duration Adherence	Type of Study	Country	Macronutrient Intake	Micronutrient Intake
González et al. (2018) [18]	n = 42 men, 31.5 y ± 11.9 ≥1 year GFD ND	Observational, transversal cohort study	Spain	High fat, specially SFA High protein, Low CHO, Low fiber, High cholesterol.	Low vitamin D and E, folate, iodine, and magnesium.
Churruca et al. (2015) [19]	n = 54 women, 34 y ± 13 Median duration of GFD = 10 years ND	Observational, transversal cohort study	Spain	Low energy intake, Low CHO, Low fiber, High fat.	Low vitamin D and E, Folate, Calcium, Iron, Magnesium, Iodine, Potassium and Selenium.
Bascuñán et al. (2019) [25]	n = 46 (43 women), 41.1 y ± 10.1 ≥1 year GFD Strict adherence 100% of participants	Randomized double bind controlled study	Italy	High fat, low CHO.	Low vitamin D, vitamin E, folate, thiamine (B1), calcium, iron, zinc, sodium and potassium.
Hopman et al. (2006) [36]	n = 132 (87 women), 16.6 y ± 4.4 Median duration of GFD = 9.6 years Strict adherence 75% of participants	Observational, transversal cohort study	Netherlands	High saturated fat, Low fiber.	Low Iron and Calcium.
Hallert et al. (2002) [43]	n = 30 (18 women), 55 y −10 years GFD Strict adherence 100% of participants	Observational, longitudinal cohort study	Sweden	Low folate.	
Wild et al. (2010) [44]	n = 93 62 women, 53 y ± 13; 31 men, 56 y ± 15 ≥6 months GFD (mean duration: 8 y) ND	Observational, longitudinal cohort study	UK	Low fiber, high sugar.	Low vitamin D, folate, calcium, iron, zinc, magnesium and manganese.
Martin et al. (2013) [45]	n = 73 (55 women), 18–80 y Median duration of GFD = 7.5 years ND	Observational, transversal cohort study	Germany	Low CHO, Low fiber.	Low vitamin B1, B2, B6, Folate, magnesium and iron.
Thompson et al. (2005) [46]	n = 47 (39 women), 51 y ± 11 Median duration of GFD = 5.3 years Strict adherence 100% of participants	Observational, transversal cohort study	USA	Low fiber.	Low iron and calcium.
Jamieson et al. (2020) [47]	n = 35 (29 women), 47 y ± 11.5 Median duration of GFD = 6.7 years ND	Observational, transversal cohort study	Canada	Low CHO, high fat, low fiber.	Low iron, calcium and vitamin C.
Ballestero-Fernández et al. (2021) [48]	n = 64 43 women, 39.17 y ± 10.62; 21 men, 38.58 y ± 9.61 ≥1 year GFD ND	Observational, transversal case-control study	Spain	Low CHO, PUFA and fiber high protein, fat and sugars.	Low folate, vitamin E, vitamin D, iodine, calcium, zinc, magnesium. Low iron in women

GFD: gluten free diet. SFA: saturated fatty acids. CHO: carbohydrates. y: years. PUFA: polyunsaturated fatty acids. ND: not determined.

**Table 3:** Summary of nutritional deficiencies in celiac adults and children.

	Celiac Adults	Celiac Children
Fat	High fat and SFA intakes	=
Carbohydrates	Low complex carbohydrate intake, but high simple sugar intake	=
Fiber	Low fiber intake	=
Vitamins	Low intakes of Vitamin D, E and B group vitamins (B1, B2, B6, B9)	Low intakes of Vitamin D and B group vitamins (B1, B2, B6, B9)
Minerals	Low intakes of iron, calcium, magnesium, zinc, iodine, potassium, selenium and manganese.	Low intakes of iron, calcium, magnesium and zinc.

SFA: saturated fatty acids. “=” symbol means that same data were found in children.

**Table 4:** Differences between celiac men and women.

	Women	Men
Macronutrients	Unbalanced distribution and intake	=
Fiber	Lower fiber intake	Low fiber intake
Vitamins	Various deficiencies	=
Minerals	Lower Fe, Ca, I and K intakes	Lower Mg intake

“=” symbol means that same data were found in men.

**Table 5:** Differences between celiac patients and healthy general population.

	Healthy Population	Celiac Patients
Energy	=	=
Fat	High fat intake	Higher fat and SFA intake
Proteins	=	=
Carbohydrates	Low carbohydrate intake	Lower complex carbohydrate intake, higher simple sugar intake
Fiber	Low intake	Lower fiber intake
Vitamins	Low folate intake	Lower vitamin E and B group vitamins intakes. Lower folate intake and higher tHcy serum levels.
Minerals		Lower magnesium, selenium, iron and zinc intake

SFA: saturated fatty acids. “=” symbol means that same data were found.

**Table 6:** Dietary guidelines for a balanced GFD.

Guideline for a Secure and Balanced GFD	Result in the Dietary Profile
Promote consumption of plant-based foods, such as fruits, vegetables, legumes, nuts and naturally gluten-free whole grain cereals and pseudocereals (quinoa, amaranth, etc.)	↓ fats, specially saturated ↓ sugars ↑ complex carbohydrates ↑ fiber ↑ vitamins (folate, riboflavin, vitamin C and E, etc.) ↑ minerals
Reduce GFP consumption.	
Fortify naturally gluten-free foods in micronutrients	↑ vitamins ↑ minerals
Fortify GFP in micronutrients and balance their macronutrient content	Helpful in the macronutrient and micronutrient balance achievement
Increase dairy products, as well as legumes and cereals	↑ iron, calcium and vitamin D
Increase vegetables, pulses and pseudocereals	↑ B group vitamins
Continuous supervision of celiac patients going on a GFD	↑ adherence to the diet

GFD: Gluten-free diet. ↓: decrease; ↑: increase.

## Supplementary Materials

The following is available online at https://www.mdpi.com/article/10.3390/nu13082877/s1, Figure S1: Schema of how articles related to “nutritional deficiencies among adults with celiac disease following a GFD” were selected.
