# Supplementary material for: Nutritional Imbalances in Adult Celiac Patients Following a Gluten-Free Diet
Source: Nutrients. 2021 Aug 21;13(8):2877. doi: 10.3390/nu13082877 (PMC8398893; doi:10.3390/nu13082877)
Supplement: Supplementary file 1 [file nutrients-13-02877-s001.zip › nutrients-1313780-supplementary.pdf]

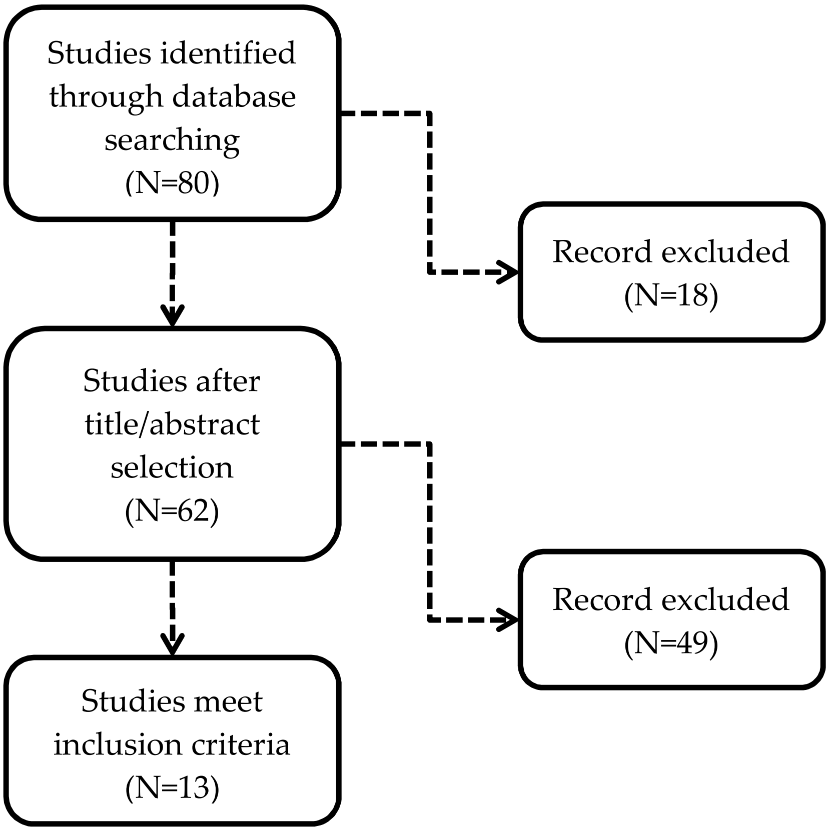

Supplementary Figure S1. Schema of how articles related to "nutritional deficiencies among adults with celiac disease following a GFD" were selected.
